# Reproductive Hormone Levels Predict Changes in Frailty Status in Community-Dwelling Older Men: European Male Ageing Study Prospective Data

**DOI:** 10.1210/jc.2017-01172

**Published:** 2017-11-24

**Authors:** Agnieszka Swiecicka, Robert J. A. H. Eendebak, Mark Lunt, Terence W. O’Neill, György Bartfai, Felipe F. Casanueva, Gianni Forti, Aleksander Giwercman, Thang S. Han, Jolanta Slowikowska-Hilczer, Michael E. J. Lean, Neil Pendleton, Margus Punab, Dirk Vanderschueren, Ilpo T. Huhtaniemi, Frederick C. W. Wu, Martin K. Rutter, on behalf of the European Male Ageing Study Group

**Affiliations:** 1 Andrology Research Unit, Division of Diabetes, Endocrinology, and Gastroenterology, School of Medical Sciences, Faculty of Biology, Medicine, and Health, University of Manchester, Manchester Academic Health Science Centre, Manchester M13 9PL, United Kingdom; 2 Arthritis Research UK Centre for Epidemiology, Faculty of Biology, Medicine, and Health, Manchester Academic Health Science Centre, University of Manchester and NIHR Musculoskeletal Biomedical Research Unit, Central Manchester University Hospitals NHS Foundation Trust, Manchester M13 9PL, United Kingdom; 3 Department of Obstetrics, Gynaecology, and Andrology, Albert Szent-Györgyi Medical University, Szeged H-6725, Hungary; 4 Department of Medicine, Santiago de Compostela University, Complejo Hospitalario Universitario de Santiago, CIBER de Fisiopatología Obesidad y Nutricion, Instituto Salud Carlos III, Santiago de Compostela 15706, Spain; 5 Sexual Medicine and Andrology Unit, Department of Experimental and Clinical Biomedical Sciences “Mario Serio,” University of Florence, Florence 50139, Italy; 6 Reproductive Medicine Centre, Malmö University Hospital, University of Lund, SE-221 00 Lund, Sweden; 7 Institute of Cardiovascular Research, Royal Holloway University of London, Egham TW20 0EX, United Kingdom; 8 Ashford and St. Peter’s NHS Foundation Trust, Surrey TW15 3AA, United Kingdom; 9 Department of Andrology and Reproductive Endocrinology, Medical University of Lodz, 91-425 Lodz, Poland; 10 Department of Human Nutrition, University of Glasgow, Glasgow G31 2ER, United Kingdom; 11 Division of Neuroscience and Experimental Psychology, School of Biological Sciences, University of Manchester, Hope Hospital, Salford M6 8HD, United Kingdom; 12 Andrology Unit, United Laboratories of Tartu University Clinics, 50406 Tartu, Estonia; 13 Department of Andrology and Endocrinology, Katholieke Universiteit Leuven, B-3001 Leuven, Belgium; 14 Department of Surgery and Cancer, Institute of Reproductive and Developmental Biology, Imperial College London, Hammersmith Campus, London W12 0HS, United Kingdom; 15 Department of Physiology, Institute of Biomedicine, University of Turku, FI-20520 Turku, Finland; 16 Manchester Diabetes Centre, Central Manchester University Hospitals NHS Foundation Trust, Manchester Academic Health Science Centre, Manchester M13 9NT, United Kingdom

## Abstract

**Context:**

Clinical sequelae of androgen deficiency share common features with frailty. Evidence supporting the role of androgens in the development of frailty is limited and conflicting.

**Objective:**

To determine associations between male reproductive hormones and prospective changes in frailty status.

**Design/Setting:**

A 4.3-year prospective cohort study of community-dwelling men participating in the European Male Ageing Study.

**Participants:**

A total of 3369 men aged 40 to 79 from eight European centers.

**Intervention:**

None.

**Main Outcome Measure:**

Frailty status was determined using frailty index (FI; n = 2278) and frailty phenotype (FP; n = 1980).

**Results:**

After adjusting for baseline frailty, age, center, and smoking, the risk of worsening FI decreased with higher testosterone (T), free T, and dihydrotestosterone (DHT) [percentage change (95% confidence interval) in FI associated with 1 standard deviation higher hormone level: –3.0 (–5.9, –1.0) for total T; –3.9 (–6.8, –2.0) for free T; and –3.9 (–6.8, –2.0) for DHT]. After further adjustment for body mass index, only free T remained a significant predictor of FI change. In fully adjusted models, higher luteinizing hormone and follicle-stimulating hormone were positively related to worsening FI only in men <60 years, and higher estradiol predicted lower likelihood of improving FP [odds ratio: 0.68 (0.52, 0.88)].

**Conclusions:**

These prospective data support the hypothesis that higher androgen levels may protect elderly men from worsening frailty. However, the causal nature of these relationships requires further investigation. Whereas raised gonadotropins in men <60 years might be an early marker of frailty, the role of estradiol in frailty needs further clarification.

## Teaser

We investigated reproductive hormones as risk factors for frailty in aging men and found significant associations of free testosterone, estradiol, and gonadotropins with changes in frailty status.

Frailty in the elderly describes a state of reduced homeostatic reserve and diminished resistance to external and internal stressors, which is associated with adverse outcomes such as disability, falls, and death (1, 2). With rising life expectancy, frailty is increasingly recognized as an important health care issue; much research has focused on investigating its etiology and natural history to help identify high-risk individuals and facilitate the development of effective prevention and treatment strategies.

The pathophysiology of frailty is poorly understood, but it has been linked with disruptions within a number of body systems, including metabolic and inflammatory pathways (3, 4). Both aging and frailty share common features in relation to changes in body composition, muscle strength, and physical function, which are accompanied by a parallel decline in androgen levels. Therefore, dysregulation within the hypothalamic-pituitary-gonadal axis has been suggested to play a role in the development of frailty. The supporting evidence, however, is rather limited. A number of studies have investigated associations of testosterone (T) with parameters of muscle function and physical performance (5, 6), but relatively few, predominantly cross-sectional, studies have focused on association between androgens and frailty (7, 8), with conflicting results. Moreover, to date, frailty (as opposed to muscle strength and physical performance) has not been studied as a clinical outcome of interventional trials of T replacement in older men.

We used the longitudinal data from the population-based European Male Ageing Study (EMAS) to determine the associations between hypothalamic-pituitary-gonadal axis hormones and change in frailty status in middle-aged and older men.

## Methods

### Subjects

Subjects included were participants in the EMAS as described previously (9). Briefly, 3369 men aged 40 to 79 were recruited between 2003 and 2005 from population-based sampling frames in eight European centers: Florence (Italy), Leuven (Belgium), Lodz (Poland), Malmö (Sweden), Manchester (United Kingdom), Santiago de Compostela (Spain), Szeged (Hungary), and Tartu (Estonia). The participants completed a series of clinical assessments and provided a fasting blood sample. Ethical approval was obtained in accordance with the local requirements.

Participants were recontacted after a minimum of 4 years (median, 4.3 years). Methods of data collection at follow-up were largely identical to the baseline study. During follow-up, 193 (6%) men died and 440 (13%) were lost to follow-up.

Participants with self-reported history of testicular, adrenal, and/or pituitary disease and/or the use of medications affecting the functioning of the hypothalamic-pituitary-gonadal axis (androgens, antiandrogens, 5-*α* reductase inhibitors, gonadotropin-releasing hormone analogs, anabolic steroids, strong opioids, and oral corticosteroids) were excluded. We did this for two reasons: 1) because we were interested in the associations of physiological, rather than pathological, differences in hormone levels with changes in frailty and 2) because the diseases causing altered hormone levels could have direct effects on frailty levels and confound the relationship between hormone levels and changes in frailty.

### Assessments

All participants were asked questions concerning lifestyle, general health, and comorbidities. The interviewer-assisted questionnaire included the Medical Outcomes Study 36-Item Short Form Survey (10), the Physical Activity Scale for the Elderly (11), Beck’s Depression Inventory (BDI) (12), and the International Prostate Symptoms Score (13). Physical function was assessed by Reuben’s Physical Performance Test (14) and the Tinetti Balance and Postural Stability Index (15). The Rey-Osterrieth Complex Figure Test (16), the Camden Topographical Recognition Memory Test (17), and the Digit-Symbol Substitution Test (18) were used to assess the cognitive function. Anthropometric parameters measured included height, weight, waist, mid upper arm, and calf circumferences, and skinfold thicknesses.

### Frailty measures

Frailty was characterized by the two commonly used approaches: frailty index (FI) and frailty phenotype (FP).

The EMAS FI comprised 39 health deficits (symptoms, signs, and functional and cognitive impairments) that accumulate with age and are associated with adverse health outcomes. These variables were derived from Medical Outcomes Study 36-Item Short Form Survey and BDI questionnaires, physical performance and cognitive test data, and self-reported comorbidities. The EMAS FI was created using a standardized procedure (19) and was calculated as the number of deficits present divided by the maximum number of deficits possible. Binary variables (coded as 0 or 1) indicated the absence or presence of a deficit, and intermediate responses (*e.g.*, sometimes/maybe) were coded as 0.5. Continuous variables were dichotomized based on the distribution of participants’ scores (cut points set at the worst performing 10th centile) (Supplemental Table 1). Individuals with >20% of deficit variables missing were excluded (Supplemental Figs. 3 and 4).

EMAS FP was adapted from the five criteria used in the Cardiovascular Health Study (2): sarcopenia, exhaustion, slowness, weakness, and low activity. Variables used to construct EMAS FP and population-specific frailty thresholds are presented in Supplemental Table 2, alongside the original Cardiovascular Health Study criteria. Individuals with three or more criteria were classed as “frail,” those with one or two as “prefrail,” and those with none as “robust” (Supplemental Fig. 5). The EMAS FP has been shown to be predictive of adverse health outcomes such as falls and death (20).

### Reproductive hormones and SHBG

A fasting morning (before 10 am) venous blood sample was taken at baseline and follow-up. A validated gas (or liquid) chromatography-mass spectrometry system was used to analyze T [intra- and interassay coefficients of variation (CVs): 2.9% and 3.4%], estradiol (E_2_; CVs: 3.5% and 3.7%), and dihydrotestosterone (DHT; CVs: 3.1% and 4.1%).

Luteinizing hormone (LH), follicle-stimulating hormone (FSH), and sex hormone–binding globulin (SHBG) were measured using the Modular E170 platform electrochemiluminescence immunoassay (Roche Diagnostics, Mannheim, Germany). Intra- and interassay CVs were 1.9% and 2.7% for LH, 0.9% and 1.9% for FSH, and 1.9% and 3.2% for SHBG.

Free T (fT) levels were derived from total T, SHBG, and albumin concentrations using Vermeulen’s formula (21).

### Statistical analysis

Descriptive statistics were presented as mean ± standard deviation (SD) or n (%), and statistical significance of between-group differences was assessed using analysis of variance.

#### FI models

In view of the right skewing of the FI variable, relationships between individual endocrine predictors and FI at follow-up (outcome) were assessed using negative binomial regression modeling. The FI metric was calibrated as an additive 0- to 39-count scale, where “0” represented no deficits and “39” represented the maximum deficits. Results were presented as percentage change [95% confidence interval (CI)] in FI associated with a 1 SD higher baseline hormone level (negative values indicating improving frailty and positive values indicating worsening frailty during follow-up).

#### FP models

Change in frailty was defined using transitions in frailty states between baseline and follow-up. The transitions considered were worsening frailty (robust or prefrail at baseline progressing to prefrail or frail at follow-up; referent category: persistent robust or persistent prefrail) and improving frailty (prefrail or frail at baseline transitioning to robust or prefrail state at follow-up; referent category: persistent frail or prefrail).

Logistic regression models determined relationships between individual predictors (hormones at baseline) and outcome (transition in FP). Each hormone was represented by an untransformed value and standardized as a *z* score [(raw score – mean)/SD]. The results were displayed as odds ratios (ORs) with 95% CIs for a 4.3-year change in frailty status associated with a 1 SD difference in baseline hormone level.

Regression models were adjusted for baseline frailty status, age, center, smoking (current or ex-/nonsmoker), and body mass index (BMI) category (BMI < 25, 25 to 30, and ≥ 30). These covariates were chosen because they correlated with predictors and were not components of the FP or FI. Analyses in which E_2_ was a predictor were adjusted for total T level—the main precursor for E_2_ production in men.

All analyses were performed using STATA 13 SE software (StataCorp LP, College Station, TX).

## Results

### Population characteristics

Of the 3369 men who participated in EMAS, 2278 men remained in the FI analysis and 1980 in the FP analysis after exclusion of those with pituitary, testicular, or adrenal disease or use of medication affecting hypothalamic-pituitary-gonadal axis (n = 312), missing FI (n = 204), or FP (n = 502) data and failure to attend for follow-up assessment (n = 575) (Supplemental Fig. 1).

Compared with the main analytical sample, men lost to follow-up (n = 407) were older and had higher systolic blood pressure and greater prevalence of smoking, depression, diabetes, and frailty at baseline (Supplemental Tables 3 and 4). This was also the case for the men who died (n = 168), with addition of a higher creatinine and waist-hip ratio in this group when compared with the analytical sample (Supplemental Tables 3 and 4).

### Baseline characteristics

Baseline clinical and hormonal characteristics of the study participants are shown in Table 1. The men had a mean ± SD age of 58 ± 11 years and a BMI of 28 ± 4 kg/m^2^. Six percent were known to suffer from diabetes, 33% reported history of a cardiovascular disease (CVD), and 21% had history of depression.

**Table 1.** **Baseline Characteristics of the Study Population**

Baseline Parameter	Mean ± SD or n (%)
N	2278
Age, y	58 ± 11
BMI, kg/m^2^	28 ± 4
Smoking, n (%)	447 (20%)
Frequent alcohol, n (%)	546 (24%)
Systolic BP, mm Hg	145 ± 20
Diastolic BP, mm Hg	87 ± 12
PASE	206 ± 87
Severe depression (BDI band 4–6), n (%)	69 (4%)
Mild depression (BDI band 2–3), n (%)	363 (17%)
CVD, n (%)	741 (33%)
Diabetes, n (%)	132 (6%)
Total T, nmol/L	16.9 ± 6.0
fT, pmol/L	303.3 ± 85.9
DHT, nmol/L	1.34 ± 0.6
E_2_, pmol/L	73.6 ± 24.6
SHBG, nmol/L	41.8 ± 19.0
FSH, IU/L	8.0 ± 8.4
LH, IU/L	6.0 ± 4.0

Abbreviations: BP, blood pressure; N, number; PASE, Physical Activity Scale for the Elderly.

Differences in baseline parameters between frailty transition groups are shown in Table 2. When compared with men who remained robust or prefrail, those whose frailty status deteriorated over follow-up (n = 426; Supplemental Fig. 2) were older and had lower baseline BMI, but higher SHBG, LH, and FSH hormones and a higher prevalence of diabetes and CVD. When compared with men who remained persistently frail or prefrail, men who experienced improvement in their frailty status (n = 196; Supplemental Fig. 2) were younger and had a lower prevalence of CVD and lower baseline E_2_, SHBG, and gonadotropin levels.

**Table 2.** **Baseline Parameters Stratified by Frailty Transition Group, as Assessed by FP Derived From the Cardiovascular Health Study**

Baseline Parameter	Worsening Frailty*^a^*	Persistent Robust and Persistent Prefrail	*P* Value	Improving Frailty*^b^*	Persistent Frail and Persistent Prefrail	*P* Value
N	426	1352		196	236	
Age, y	60 ± 11	57 ± 10	<0.001	59 ± 10	64 ± 10	<0.001
BMI, kg/m^2^	27.3 ± 3.9	27.7 ± 4.0	0.025	27.3 ± 3.7	27.2 ± 5.1	0.322
Smoking, n (%)	95 (22)	257 (19)	0.145	42 (22)	54 (23)	0.771
Frequent alcohol, n (%)	114 (27)	317 (24)	0.174	48 (25)	49 (21)	0.378
Systolic BP, mm Hg	145 ± 20	145 ± 20	0.910	144 ± 21	147 ± 22	0.158
Diastolic BP, mm Hg	87 ± 11	88 ± 12	0.297	89 ± 13	86 ± 12	0.063
PASE	192 ± 80	217 ± 86	<0.001	158 ± 93	145 ± 94	0.124
Severe depression (BDI band 4–6), n (%)	7 (2)	33 (3)	0.399	8 (5)	18 (11)	0.092
Mild depression (BDI band 2–3), n (%)	77 (19)	194 (15)	0.064	47 (25)	65 (30)	0.280
CVD, n (%)	154 (36)	364 (29)	0.006	68 (35)	112 (48)	0.007
Diabetes, n (%)	32 (8)	63 (5)	0.024	10 (5)	22 (9)	0.097
Total T, nmol/L	17.2 ± 6.1	17.0 ± 6.0	0.363	16.4 ± 5.8	17.3 ± 6.5	0.179
fT, pmol/L	300.1 ± 83.0	310.3 ± 86.3	0.076	290.1 ± 79.7	285.2 ± 88.9	0.454
DHT, nmol/L	1.37 ± 0.6	1.34 ± 0.6	0.221	1.31 ± 0.5	1.37 ± 0.6	0.669
E_2_, pmol/L	74.5 ± 25.8	73.8 ± 24.0	0.668	69.8 ± 21.3	77.5 ± 29.8	0.010
SHBG, mmol/L	43.6 ± 18.7	40.4 ± 17.9	<0.001	42.3 ± 20.0	47.5 ± 19.5	0.002
FSH, IU/L	8.4 ± 7.8	7.3 ± 6.9	0.003	8.0 ± 8.9	9.9 ± 12.1	0.017
LH, IU/L	6.1 ± 3.6	5.6 ± 3.5	0.010	5.7 ± 3.9	6.9 ± 5.5	0.005

Data are expressed as mean ± SD for continuous variables or as number (percentage) for binary categorical variables. *P* values were calculated using baseline parameters and using analysis of variance.

Abbreviations: BP, blood pressure; N, number; PASE, Physical Activity Scale for the Elderly.

^a^ Worsening Frailty = robust or prefrail men at baseline progressing to prefrail or frail state at follow-up.

^b^ Improving Frailty = prefrail or frail men at baseline transitioning to robust or prefrail state at follow-up.

### Hormonal predictors of worsening frailty

#### FI

In models adjusted for baseline frailty, age, center, and smoking status, higher baseline levels of total T, free T (fT), and DHT were associated with a lower likelihood of worsening FI (Table 3). After additional adjustment for BMI, only fT remained a significant predictor of change in FI. Higher baseline levels of SHBG, FSH, and LH were associated with higher risk of worsening FI in models adjusted for baseline FI (Table 3), but age adjustment attenuated these relationships. Higher E_2_ levels predicted worsening FI in a model adjusted for baseline FI, age, center, and smoking; however, the statistical significance was lost after additional adjustment for BMI (Table 3).

**Table 3.** **Relationship Between Baseline Level of Endocrine Predictors and a 4-Year Percentage Change in FI**

Models and Adjustments													
Baseline Parameter	N	Model 1 Baseline Frailty			Model 2 Baseline Frailty and Age			Model 3 Baseline Frailty, Age, Center, Smoking			Model 4 Baseline Frailty, Age, Center, Smoking, BMIc		
		% Change^*a*^	95% CI	*P* Value	% Change*^a^*	95% CI	*P* Value	% Change*^a^*	95% CI	*P* Value	% Change*^a^*	95% CI	*P* Value
Total T	2262	−3.0	−4.9, –0.4	0.020	−3.0	−4.9, –0.5	0.015	−3.0	−5.9, –1.0	0.004	−1.0	−3.0, 1.0	0.354
fT	2257	−8.6	−10.5, –5.9	<0.001	−4.9	−7.7, –3.0	<0.001	−3.9	−6.8, –2.0	0.001	−2.8	−4.9, –0.3	0.030
DHT	2255	−3.0	−4.9, –0.6	0.013	−3.9	−6.8, –2.0	<0.001	−3.9	−6.8, –2.0	<0.001	−2.0	−4.0, 0.4	0.105
E_2_*^b^*	2254	1.0	−1.0, 3.0	0.389	2.0	−1.0, 4.0	0.133	3.0	−1.0, 5.1	0.027	1.0	−1.0, 4.1	0.407
SHBG	2268	5.1	2.0, 7.2	<0.001	0.4	−2.0, 2.0	0.721	−1.1	−3.0, 1.3	0.368	1.0	−1.0, 3.0	0.391
FSH	2267	5.1	3.0, 7.2	<0.001	2.0	−0.3, 4.0	0.091	1.0	−1.0, 3.1	0.311	1.0	−0.9, 3.0	0.274
LH	2266	4.1	2.0, 6.0	<0.001	2.0	−0.5, 7.0	0.113	1.0	−1.0, 3.0	0.285	1.0	−0.5, 3.0	0.138

Abbreviations: BMIc, BMI categories (<25, 25 to 30, ≥30); N, sample size.

^a^ Change (percentage change/4 years) in FI per SD increase in hormone level. Negative percentage change means that the baseline hormone level is associated with improvement of frailty status, and positive percentage change means that the hormone is associated with worsening frailty status.

^b^ Models 2 to 4 additionally adjusted for baseline total T level.

#### FP

In keeping with the FI results, higher baseline fT levels were associated with a lower likelihood of worsening FP, but this association became statistically nonsignificant after adjusting for age (Table 4). Also in keeping with FI data, higher baseline SHBG, LH, and FSH levels were significantly associated with worsening FP in models adjusted for baseline FP, but age adjustment, again, attenuated these relationships. Baseline levels of T and DHT were not related to worsening FP in any model.

**Table 4.** **Multivariable-Adjusted OR (95% CI) for Worsening FP Associated With Baseline Endocrine Predictors**

Models and Adjustments													
Baseline Parameter	N	Model 1 Baseline Frailty			Model 2 Baseline Frailty and Age			Model 3 Baseline Frailty, Age, Center, Smoking			Model 4 Baseline Frailty, Age, Center, Smoking, BMIc		
		OR	95% CI	*P* Value	OR	95% CI	*P* Value	OR	95% CI	*P* Value	OR	95% CI	*P* Value
Total T	1766	1.04	0.93, 1.16	0.446	1.04	0.93, 1.16	0.520	1.08	0.96, 1.22	0.187	1.05	0.92, 1.18	0.474
fT	1760	0.86	0.77, 0.96	0.008	0.98	0.87, 1.1	0.702	1.03	0.91, 1.16	0.681	0.99	0.88, 0.04	0.979
DHT	1759	1.07	0.96, 1.19	0.213	1.03	0.92, 1.15	0.601	1.01	0.90, 1.14	0.822	0.96	0.85, 1.09	0.574
E_2_*^a^*	1759	1.04	0.94, 1.16	0.435	0.99	0.87, 1.13	0.894	1.07	0.94, 1.23	0.300	1.11	0.97, 1.28	0.136
SHBG	1769	1.25	1.12, 1.39	<0.001	1.08	0.96, 1.22	0.181	1.10	0.97, 1.24	0.144	1.06	0.94, 1.21	0.342
FSH	1768	1.21	1.09, 1.34	<0.001	1.09	0.98, 1.22	0.123	1.11	0.99, 1.24	0.085	1.10	0.98, 1.24	0.094
LH	1767	1.20	1.08, 1.33	0.001	1.09	0.97, 1.22	0.132	1.08	0.96, 0.21	0.183	1.07	0.95, 1.20	0.257

Abbreviations: BMIc, BMI categories (<25, 25 to 30, ≥30); DM, diabetes mellitus; N, sample size.

^a^ Models 2 to 4 additionally adjusted for baseline total T level.

### Hormonal predictors of improving frailty

In prefrail or frail men, higher baseline levels of E_2_ were associated with a lower likelihood of improvement in FP at follow-up in the fully adjusted model (Table 5). Higher levels of SHBG and LH were associated with a lower likelihood of improving FP, but these associations became statistically nonsignificant after adjusting for age. Levels of T, fT, and DHT were not related to FP improvement in any model.

**Table 5.** **Multivariable-Adjusted OR (95% CI) for Improving FP Associated With Baseline Endocrine Predictors**

Models and Adjustments													
Baseline Parameter	N	Model 1 Baseline Frailty			Model 2 Baseline Frailty and Age			Model 3 Baseline Frailty, Age, Center, Smoking			Model 4 Baseline Frailty, Age, Center, Smoking, BMIc		
		OR	95% CI	*P* Value	OR	95% CI	*P* Value	OR	95% CI	*P* Value	OR	95% CI	*P* Value
Total T	427	0.88	0.73, 1.06	0.181	0.85	0.69, 1.03	0.107	0.87	0.70, 1.07	0.183	0.87	0.69, 1.09	0.215
fT	427	1.09	0.90, 1.32	0.385	0.89	0.72, 1.10	0.290	0.89	0.71, 1.11	0.319	0.90	0.71, 1.13	0.349
DHT	428	0.91	0.75, 1.10	0.322	0.92	0.75, 1.12	0.413	0.94	0.76, 1.16	0.589	0.96	0.77, 1.21	0.764
E_2_*^a^*	428	0.74	0.61, 0.89	0.002	0.81	0.64, 1.01	0.061	0.71	0.56, 0.91	0.008	0.68	0.52, 0.88	0.004
SHBG	429	0.76	0.63, 0.92	0.006	0.88	0.72, 1.08	0.223	0.90	0.72, 1.12	0.343	0.91	0.72, 1.15	0.446
FSH	429	0.88	0.76, 1.02	0.095	0.99	0.85, 1.15	0.906	1.02	0.88, 1.18	0.804	1.02	0.88, 1.19	0.791
LH	428	0.80	0.67, 0.96	0.018	0.91	0.76, 1.09	0.302	0.94	0.79, 1.12	0.484	0.94	0.79, 1.12	0.492

Abbreviations: BMIc, BMI categories (<25, 25 to 30, ≥30); DM, diabetes mellitus; N, sample size.

^a^ Models 2 to 4 additionally adjusted for baseline total T level.

### Secondary analyses

In a secondary analysis, age modified the association between gonadotropins and FI. In the fully adjusted models, higher LH and FSH levels were related to worsening FI in younger (<60 years old) but not older men [percentage change in FI associated with 1 SD higher hormone level: FSH, 10% (*P* = 0.002); LH, 9.6% (*P* = 0.003)].

In FP models, further adjustment for CVD, depression (BDI score), and diabetes did not alter the relationship between hormones and FP (Supplemental Tables 6 and 7); the association between E_2_ and improvement in FP persisted [OR, 0.68 (0.51, 0.89); *P* = 0.005]. FI models were not adjusted for CVD or diabetes because they are components of FI.

## Discussion

### Main findings

These data describe the longitudinal associations of reproductive hormone levels with changes in frailty status in middle-aged and elderly men using two different frailty models. Our main findings were (1) higher fT levels were associated with a lower risk of worsening frailty status with consistent directions of association using two different frailty constructs (FI and FP); (2) in age-adjusted FI models, higher androgen levels (fT, T, and DHT) remained significantly associated with improving frailty status, suggesting that these relationships cannot be explained by age-related differences in androgen levels; (3) we provided evidence that frailty status was less likely to improve in prefrail or frail men with higher baseline E_2_ levels; and (4) we showed that higher LH and FSH levels were associated with worsening frailty status, but that these relationships were largely explained by age-related changes in these hormones, except in younger men.

We have previously shown that nonandrogenic anabolic hormones such as IGF-1, IGFBP3, and vitamin D were independently associated with change in frailty status in middle-aged and older men (22). These two complimentary reports enhance our understanding of a relative role of the endocrine system in the development of frailty and suggest that there may be multiple underlying hormonal mechanisms involved in the pathogenesis of frailty.

### Prior studies

This is, to our knowledge, the first study to simultaneously use two common frailty measures to investigate relationships between reproductive hormones and frailty. Furthermore, whereas the majority of frailty studies have been cross-sectional and have focused on elderly men, we investigated frailty prospectively and included younger men (40 to 59 years) to seek evidence that hormonal levels might predispose to the development of onset of frailty.

Our results contrast with those of the Concord Health and Ageing in Men Project (23), where 1166 men >70 years of age were followed for 2.1 years. The authors found no association between baseline levels of T, fT, DHT, SHBG, or gonadotropins and worsening frailty. Moreover, the authors observed that lower levels of estrone (but not E_2_) were linked to prevalent and incident frailty, but these findings were not confirmed when an alternative measure of physical frailty (Study of Osteoporotic Fractures FI) was used (23). Methodological differences (studying much older subjects, having shorter follow-up, smaller sample size, and not adjusting for baseline frailty status) may have contributed to differences compared with our study.

Cawthon *et al*. (8) reported longitudinal associations between lower baseline bioavailable T, but not T, E_2_, or SHBG, and a higher risk of frailty at 4.1 years in 1245 men aged >65 years participating in the Osteoporotic Fractures in Men Study. Free T was not evaluated. Adjustment for a number of covariates, including age, baseline frailty, BMI, comorbidities, education, smoking status, marital status, and self-rated health, attenuated this association. This loss of statistical significance could perhaps be explained by covariates such as BMI being on the causal pathway linking hormone levels to frailty. However, in our study, fT and E_2_ remained significantly predictive of frailty changes after covariate adjustments. Therefore, overadjustment bias is unlikely to account for the discrepant results. Notwithstanding the differences between bioavailable T and fT, it is possible that sample size, the older age range (>65 years), and the quartile modeling methodology used by Cawthon *et al*. (8) may have mitigated against finding persistently significant relationships.

By contrast, the Health in Men Study reported statistically significant associations between baseline levels of immunoassayed T and calculated fT as well as LH and frailty measured by the FRAIL scale in 1586 men aged 70 to 88 years followed for 6 years (7). Following adjustment for age, BMI, smoking, diabetes, social support, and impaired hearing and vision, only fT remained significantly correlated with follow-up frailty status. These findings are consistent with our present results. Our study adds important new data by assessing frailty through objective and validated clinical assessments (not only questionnaires) and by assessing participants using two frailty measures in a larger and younger cohort that captures earlier frailty transitions.

### FI vs FP

Although we have shown significant associations between reproductive hormones and frailty, it is important to note that relationships varied between the two different constructs of frailty assessment. Covariates, mainly age and BMI, confounded and attenuated the hormone-frailty relationships, especially those between hormones and FP. The discrepancies in the statistical significance of the results could be explained by two factors. First, the results might reflect differences in the frailty definitions used in each model; whereas FP is a measure of a physical frailty, FI is more holistic and incorporates physical, psychological, and cognitive factors. Therefore, although higher androgens were related to lower risk of worsening FI, the lack of significant associations between androgens and worsening FP might indicate that these hormones are more strongly predictive of general health rather than musculoskeletal function. Alternatively, differences in statistical modeling of “change” in frailty may explain the inconsistent associations with T and gonadotropins. FI, as a continuous measure of frailty, offers a relatively greater sensitivity to detect changes in frailty status compared with the less frequent transitions between FP categories.

### Potential pathophysiological mechanisms

Decline in muscle mass and function is thought to be central to the development of frailty, and a large body of evidence strongly supports the important anabolic role of T on the skeletal muscle. T stimulates muscle fiber hypertrophy through its action on muscle protein synthesis and inhibition of degradation pathways (24); nonetheless, the associations between T and measurements of muscle strength and physical performance remain inconsistent (25, 26). Additionally, low T is thought to be proinflammatory, which has been liked to frailty development (27).

Although E_2_ is thought to be related to adiposity rather than muscle function in men (28), negative associations between E_2_ and muscle mass and strength have been reported (29, 30). E_2_ might therefore be linked to frailty through obesity or sarcopenic obesity, a feature of frailty associated with poorer outcomes. We confirmed that the effects of E_2_ on frailty were independent of T level. Because E_2_ displays proinflammatory properties *in vivo* (31), indirect effects of E_2_ on frailty via disturbance in inflammatory pathways need to be considered. Nonetheless, further adjustment for baseline levels of high-sensitivity C-reactive protein and leptin levels did not change the associations between E_2_ and improving FP [OR, 0.7 (0.52, 0.95); *P* = 0.022]. The potential pathophysiological mechanisms linking E_2_ and frailty remain poorly understood and require further investigation.

### Strengths and limitations

This study has several strengths including: (1) use of a well-defined longitudinal, community-based cohort; (2) having a large sample size with adequate power to provide conclusive results; (3) use of standardized methods in central laboratories to assess hormone levels, including mass spectrometry; (4) use of two well-validated frailty models; and (5) the inclusion of men under 65 years of age who have not been studied previously in this context.

We acknowledge some limitations: (1) The response rate for participation was 41%. Although this is comparable to other large epidemiological studies, the occurrence of frailty in the study might have been over- or underestimated through selection. (2) One hundred ninety-three men died during follow-up, and 440 men were lost to follow-up. Therefore, the true incidence of frailty has probably been underestimated. Because this would bias the results toward the null, the reported strength of our associations are likely to be conservative. (3) Our analysis is based on the results of single hormone measurements, which do not capture pulsatile hormone variation and could attenuate regression coefficients toward the null through regression dilution bias.

## Conclusion

In summary, these prospective data provide important insight into the potential role of reproductive hormones in the development, progression, and recovery of frailty in aging men. The results cannot confirm a causal relationship between androgen status and progression of frailty, but the clear associations shown here make a strong case for definitive, large interventional trials of T therapy in frail men to determine whether such treatment would be beneficial. We show that raised gonadotropins in men <60 years old might be an early marker of frailty and accelerated aging and suggest that the role of E_2_ in frailty requires further investigation.
