# Supplementary material for: Reproductive Hormone Levels Predict Changes in Frailty Status in Community-Dwelling Older Men: European Male Ageing Study Prospective Data
Source: J Clin Endocrinol Metab. 2017 Nov 24;103(2):701–9. doi: 10.1210/jc.2017-01172 (PMC5800832; doi:10.1210/jc.2017-01172)

| **Supplemental Table 1. The list of health deficit variables comprising 39-item EMAS Frailty Index** | | |
| --- | --- | --- |
| **Origin** | **Variable/ Item** | **Score** |
| 36-Item Short Form Survey | Self-rated general health | Fair/ Poor =1, Good=0.5, Excellent/ Very good=0 |
|  | Feeding yourself | Limited / Limited a little=1, Not Limited=0 |
|  | Walking in your home | Limited / Limited a little=1, Not Limited=0 |
|  | Bathing and dressing yourself | Limited / Limited a little=1, Not Limited=0 |
|  | Walking 1 km | Limited =1, Limited a little=0.5, Not Limited=0 |
|  | Walking more than 1 km | Limited =1, Limited a little=0.5, Not Limited=0 |
|  | Climbing one flight of stairs | Limited =1, Limited a little=0.5, Not Limited=0 |
|  | Climbing several flights of stairs | Limited =1, Limited a little=0.5, Not Limited=0 |
|  | Ability to perform moderate activity | Limited =1, Limited a little=0.5, Not Limited=0 |
|  | Ability to perform vigorous activity | Limited =1, Limited a little=0.5, Not Limited=0 |
|  | In the past 4 weeks have you accomplished less than you would like as a result of your physical health | All/Most of time=1, Sometime=0.5, Little time/ None=0 |
|  | In the past 4 weeks have you cut down on the amount of time spent on work or other activities as a result of emotional problems | All/Most of time=1, Sometime=0.5, Little time/ None=0 |
|  | In the past 4 weeks, have you been feeling full of life | Little time/ None=1, Sometime=0.5, All/Most of time=0 |
|  | In the past 4 weeks, have you been feeling 'in the dumps' | All/Most of time=1, Sometime=0.5, Little time/ None=0 |
|  | In the past 4 weeks, have you been feeling downhearted | All/Most of time=1, Sometime=0.5, Little time/ None=0 |
|  | In the past 4 weeks, have you been feeling tired | All/Most of time=1, Sometime=0.5, Little time/ None=0 |
|  | During the past 6 months, have you experienced serious illness or injury to yourself | Yes=1, No=0 |
| Beck Depression Inventory | Change in sleep pattern | Sleeping less/ lot more=1, Sleeping more/same=0 |
|  | Concentration | Poor=1, Fair=0.5, As good as ever=0 |
| International Prostate Symptom Score | Over the past month, how often have you had to: |  |
|  | Postpone urination | Always,>50%=1, about or less than 50%=0.5, Not at all, <20%=0 |
|  | Night urinate | twice or more =1, once or not at all =0 |
|  | Weak Stream | Always,>50%=1, about or less than 50%=0.5, Not at all, <20%=0 |
| Self-reported morbidities | Heart condition | Yes=1, No=0 |
|  | High blood pressure | Yes=1, No=0 |
|  | Bronchitis | Yes=1, No=0 |
|  | Asthma | Yes=1, No=0 |
|  | Diabetes | Yes=1, No=0 |
|  | Liver condition | Yes=1, No=0 |
|  | Kidney condition | Yes=1, No=0 |
|  | Prostate disorder | Yes=1, No=0 |
|  | Thyroid disorder | Yes=1, No=0 |
|  | Cancer (ever) | Yes=1, No=0 |
|  | Stroke (ever) | Yes=1, No=0 |
| Rey-Osterrieth Complex Figure (ROCF) | Copying | Score <28, =1, Score≥ 28, = 0 |
|  | Delayed reproduction | Score <8, =1, Score ≥ 8, = 0 |
| Camden Topographical Recognition Memory (CTRM) | Total score | Score <16, =1, Score≥ 16, = 0 |
| Digit-Symbol Substitution (DSST) test | Total score | Score <16, =1, Score≥ 16, = 0 |
| Physical Performance Test | Time to walk 15.4 meters | Time≥ 16.7, =1, Time < 16.7, = 0 |
| Tinetti test | Total score | Score < 25, =1, Score ≥25, = 0 |

| **Supplemental Table 2. The European Male Ageing Study frailty phenotype criteria and the original Cardiovascular Health Study criteria** | | | | | | |
| --- | --- | --- | --- | --- | --- | --- |
|  | | | | | | |
| **Criteria** |  | **Cardiovascular Health Study** | |  | **European Male Ageing Study** | |
|  |  |  |  |  |  |  |
|  |  |  |  |  |  |  |
|  |  | **Adopted Measure** | **Cut-point** |  | **Adopted Measure** | **Cut-point** |
|  |  |  |  |  |  |  |
| ***Exhaustion/*** |  | Centre for Epidemiological Studies Depression Scale; | Answered ' Most of the time' or ' A moderate amount of the time' |  | Beck Depression Inventory | I don't have enough energy to do very much/anything' or |
| ***Poor endurance*** |  | Question 'I felt that everything I did was an effort' or 'I could not get going' |  |  |  | I am too tired to do a lot of/ most of the things I used to' |
|  |  |  |  |  |  |  |
|  |  |  |  |  |  |  |
| ***Slowness*** |  | Walking time: 15 feet | Slowest 20% by gender and height |  | Walking time: 50 feet | Slowest height-adjusted quintile of participants aged>65 years: |
|  |  |  |  |  |  | ≥17.2 sec for height ≤173.5cm; ≥16 sec for height >173.5cm; |
|  |  |  |  |  |  |  |
|  |  |  |  |  |  |  |
| ***Low activity*** |  | Minnesota Leisure Time Activity questionnaire; | Lowest 20% |  | Physical Activity Scale for the Elderly | Lowest quintile of participant aged > 65 years: score ≤78 |
|  |  | Kilocalorie expenditure per week |  |  |  |  |
|  |  |  |  |  |  |  |
|  |  |  |  |  |  |  |
| ***Weakness*** |  | Grip strength | Lowest 20% by gender and BMI |  | 5 Chair-stands test | Slowest 10% of participants aged >65: ≥17.8 sec or unable |
|  |  |  |  |  |  |  |
|  |  |  |  |  |  |  |
| ***Sarcopenia*** |  | Weight loss | > 10 pounds unintentional weight loss in the past 12 months |  | Mid-upper arm muscle circumference | Lowest 10% of participants aged >65 years: ≤ 23.7 cm |
|  |  |  |  |  |  |  |

| **Supplemental Table 3. Baseline Characteristics of Participants, men with incomplete data on frailty phenotype at either phase, those were lost to follow-up and men who died during follow-up.** | | | | | |
| --- | --- | --- | --- | --- | --- |
|  | **Status at follow-up** | | | | |
| **Baseline parameter** | **Participant** | **Incomplete data on frailty** | **Lost to follow-up** | **Died** | **p value** |
| N | 1980 | 502 | 407 | 168 |  |
| **Age, years** | 58±10 | 61±11a | 60±12a | 69±8abc | <.001 |
| BMI, kg/m2 | 27.6±4.0 | 27.6±3.7 | 27.8±4.3 | 27.5±5.0 | 0.310 |
| **WHR** | 0.98±0.06 | 0.99±0.06 | 0.99±0.06 | 1.0±0.06a | 0.001 |
| **Smoking, n (%)** | 396(20) | 102(21) | 110(27) | 49(30) | 0.001 |
| Frequent Alcohol, n (%) | 481(24) | 116(23) | 89(22) | 30(18) | 0.300 |
| **Below degree education, n (%)** | 1398(71) | 351(70) | 199(50) | 87(55) | <.001 |
| **Systolic BP, mmHg** | 145±20 | 146±21 | 148±22a | 152±24ab | <.001 |
| Diastolic BP, mmHg | 87±12 | 87±12 | 88±13 | 86±15 | 0.386 |
| **Creatinine, μmol/L** | 92±29 | 92±21 | 95±57 | 104±75abc | 0.004 |
| **Mild depression (BDI band 2-3), n (%)** | 321(17) | 79(18) | 81(22) | 42(30) | <.001 |
| **Severe depression (BDI band 4-6), n (%)** | 50(3) | 25(6) | 22(7) | 20(17) | <.001 |
| **Cardiovascular disease, n (%)** | 620(31) | 172(34) | 141(35) | 98(59) | <.001 |
| **Diabetes, n (%)** | 106(5) | 42(9) | 36(9) | 28(17) | <.001 |
| Total Testosterone, nmol/L | 17.0±6.0 | 16.8±5.8 | 16.9±6.3 | 16.7±6.8 | 0.842 |
| **Free Testosterone, pmol/L** | 305.9±85.2 | 293.1±89.1a | 296.9±90.0 | 259.7±92.4abc | <.001 |
| Dihydrotestosterone, nmol/L | 1.34±0.6 | 1.32±0.6 | 1.33±0.6 | 1.39±0.8 | 0.824 |
| Estradiol, pmol/L | 73.7±24.3 | 72.3±25.7 | 75.3±25.9 | 77.0±30.2 | 0.111 |
| **SHBG, nmol/L** | 41.3±18.4 | 44.0±20.6a | 43.2±20.2 | 53.3±25.7abc | <.001 |
| **FSH, IU/L** | 7.6±7.3 | 9.3±11.0a | 9.2±9.1a | 13.3±14.6abc | <.001 |
| **LH, IU/L** | 5.7±3.6 | 6.4±5.1a | 6.6±4.1a | 8.6±7.9abc | <.001 |
| **Frailty phenotype: Robust** | 1512(76) | 201(71) | 240(66) | 60(41) |  |
| **Prefrail** | 449(23) | 73(26) | 107(30) | 69(48) | <.001 |
| **Frail** | 19(1) | 8(3) | 14(4) | 16(11) |  |
| **Sarcopenia** | 93(5) | 22(5) | 28(7) | 21(13) | <.001 |
| **Exhaustion** | 115(6) | 46(10) | 41(10) | 32(19) | <.001 |
| **Low activity** | 93(5) | 22(5) | 28(7) | 21(13) | <.001 |
| **Weakness** | 104(5) | 28(6) | 33(9) | 21(13) | <.001 |
| **Slowness** | 122(6) | 56(12) | 46(12) | 57(35) | <.001 |
| Data are expressed as mean ± standard deviation for continuous variables or as number (percentage) for binary categorical variables. P values were calculated using analyses of variance or the Kruskall Wallis test for continuous variables and the chi squared test for categorical variables.  a Data differ significantly (p<0.05) from those in the participant group on post-hoc analysis using Tukey-Kramer for continuous variables or the z-test for categorical variables with correction for multiple pairwise comparisons  b Data differ significantly (p<0.05) from those with incomplete data on frailty on post-hoc analysis using Tukey-Kramer for continuous variables or the z-test for categorical variables with correction for multiple pairwise comparisons.  c Data differ significantly (p<0.05) from those in the lost to follow-up group on post-hoc analysis using Tukey-Kramer for continuous variables or the z-test for categorical variables with correction for multiple pairwise comparisons.  Abbreviations: BMI, body mass index; BP, Blood Pressure; BDI, Beck Depression Inventory; FSH, follicle stimulating hormone; LH, luteinising hormone; N, number; SHBG, sex hormone binding globulin; WHR, Waist to Hip Ratio | | | | | |

| **Supplemental Table 4. Baseline Characteristics of Participants, men with incomplete data on frailty index at either phase, those were lost to follow-up and men who died during follow-up.** | | | | | |
| --- | --- | --- | --- | --- | --- |
|  | **Status at follow-up** | | | | |
| **Baseline parameter** | **Participant** | **Incomplete data on frailty** | **Lost to follow-up** | **Died** | **p value** |
| N | 2278 | 204 | 407 | 168 |  |
| **Age, years** | 58±11 | 59±11 | 60±12a | 69±8abc | <.001 |
| BMI, kg/m2 | 27.6±3.9 | 27.8±4.0 | 27.8±4.3 | 27.5±5.0 | 0.268 |
| **WHR** | 0.98±0.06 | 0.98±0.06 | 0.99±0.06 | 1.0±0.06ab | 0.002 |
| **Smoking, n (%)** | 447(20) | 51(25) | 110(27) | 49(30) | 0.001 |
| Frequent Alcohol, n (%) | 546(24) | 51(25) | 89(22) | 30(18) | 0.311 |
| **Below degree education, n (%)** | 1602(70) | 147(72) | 199(50) | 87(55) | <.001 |
| **Systolic BP, mmHg** | 145±20 | 144±22 | 148±22a | 152±24ab | <.001 |
| Diastolic BP, mmHg | 87±12 | 87±13 | 88±13 | 86±15 | 0.483 |
| **Creatinine, μmol/L** | 92±29 | 92±16 | 95±57 | 104±75abc | 0.005 |
| **Mild depression (BDI band 2-3), n (%)** | 6±6 | 7±6 | 8±7a | 11±9abc | <.001 |
| **Severe depression (BDI band 4-6), n (%)** | 363(17) | 37(21) | 81(22) | 42(29) | <.001 |
| **Cardiovascular disease, n (%)** | 69(4) | 6(4) | 22(7) | 20(17) | <.001 |
| **Diabetes, n (%)** | 741(33) | 51(25) | 141(35) | 98(59) | <.001 |
| Total Testosterone, nmol/L | 16.9±6.0 | 17.1±5.7 | 16.9±6.3 | 16.7±6.8 | 0.867 |
| **Free Testosterone, pmol/L** | 303.3±85.9 | 303.8±89.0 | 296.9±90.0 | 259.7±92.4abc | <.001 |
| Dihydrotestosterone, nmol/L | 1.34±0.6 | 1.32±0.6 | 1.33±0.6 | 1.39±0.8 | 0.997 |
| Estradiol, pmol/L | 73.6±24.6 | 71.8±24.5 | 75.5±25.6 | 77.5±29.7 | 0.107 |
| **SHBG, nmol/L** | 41.8±19.0 | 42.4±17.6a | 43.2±20.2 | 53.3±25.7abc | <.001 |
| **FSH, IU/L** | 8.0±8.4 | 7.9±6.0 | 9.2±9.1 | 13.3±14.6abc | <.001 |
| **LH, IU/L** | 5.9±4.0 | 6.0±2.7 | 6.6±4.1a | 8.6±7.9abc | <.001 |
| **Frailty index** | 0.11±0.1 | 0.12±0.09 | 0.15±0.12ab | 0.25±0.14abc | <.001 |
| Data are expressed as mean ± standard deviation for continuous variables or as number (percentage) for binary categorical variables. P values were calculated using analyses of variance or the Kruskall Wallis test for continuous variables and the chi squared test for categorical variables.  a Data differ significantly (p<0.05) from those in the participant group on post-hoc analysis using Tukey-Kramer for continuous variables or the z-test for categorical variables with correction for multiple pairwise comparisons  b Data differ significantly (p<0.05) from those with incomplete data on frailty on post-hoc analysis using Tukey-Kramer for continuous variables or the z-test for categorical variables with correction for multiple pairwise comparisons.  c Data differ significantly (p<0.05) from those in the lost to follow-up group on post-hoc analysis using Tukey-Kramer for continuous variables or the z-test for categorical variables with correction for multiple pairwise comparisons.  Abbreviations: BMI, body mass index; BP, Blood Pressure; BDI, Beck Depression Inventory; FSH, follicle stimulating hormone; LH, luteinising hormone; N, number; SHBG, sex hormone binding globulin; WHR, Waist to Hip Ratio | | | | | |

| **Supplemental Table 5. Relationship between baseline level of endocrine predictors and a 4-year % change in frailty index** | | | | | | | | | | | | | | | | | |
| --- | --- | --- | --- | --- | --- | --- | --- | --- | --- | --- | --- | --- | --- | --- | --- | --- | --- |
| Models and adjustments | | | | | | | | | | | | | | | | | |
| Baseline Parameter | N | Model 1  Baseline frailty | | | Model 2  Baseline frailty and age | | | Model 3  Baseline frailty, age, centre, smoking | | | | Model 4  Baseline frailty, age, centre, smoking, BMIc | | | Model 5  Model 4+BDI | | |
|  |  | **% change^a^** | **95% CI** | **P-value** | **% change^a^** | **95% CI** | **P-value** | **% change^a^** | **95% CI** | **P-value** | **% change^a^** | | **95% CI** | **P-value** | **% change^a^** | **95% CI** | **P-value** |
| Total Testosterone | 2262 | -3.0 | -4.9, -0.4 | 0.020 | -3.0 | -4.9, -0.5 | 0.015 | -3.0 | -5.9, -1.0 | 0.004 | -1.0 | | -3.0, 1.0 | 0.354 | -1.3 | -3.7, 1.0 | 0.277 |
| **Free Testosterone** | 2257 | -8.6 | -10.5, -5.9 | <.001 | -4.9 | -7.7, -3.0 | <.001 | -3.9 | -6.8, -2.0 | 0.001 | -2.8 | | -4.9, -0.3 | 0.030 | -3.1 | -5.6, 0.5 | 0.018 |
| DHT | 2255 | -3.0 | -4.9, -0.6 | 0.013 | -3.9 | -6.8, -2.0 | <.001 | -3.9 | -6.8, -2.0 | <.001 | -2.0 | | -4.0, 0.4 | 0.105 | -2.2 | -4.6, 0.2 | 0.071 |
| Estradiol* | 2254 | 1.0 | -1.0, 3.0 | 0.389 | 2.0 | -1.0, 4.0 | 0.133 | 3.0 | -1.0, 5.1 | 0.027 | 1.0  1.0 | | -1.0, 4.1 | 0.407 | -1.4 | 1.2, 4.1 | 0.302 |
| SHBG | 2268 | 5.1 | 2.0, 7.2 | <.001 | 0.4 | -2.0, 2.0 | 0.721 | -1.1 | -3.0, 1.3 | 0.368 | 1.0 | | -1.0, 3.0 | 0.391 | 1.2 | -1.3, 3.8 | 0.340 |
| FSH | 2267 | 5.1 | 3.0, 7.2 | <.001 | 2.0 | -0.3, 4.0 | 0.091 | 1.0 | -1.0, 3.1 | 0.311 | 1.0 | | -0.9, 3.0 | 0.274 | 1.9 | -0.2, 4.0 | 0.075 |
| **LH** | 2266 | 4.1 | 2.0, 6.0 | <.001 | 2.0 | -0.5, 7.0 | 0.113 | 1.0 | -1.0, 3.0 | 0.285 | 1.0 | | -0.5, 3.0 | 0.138 | 2.1 | 0.1, 4.2 | 0.039 |
| * Models 2-5 additionally adjusted for baseline total testosterone level  Abbreviations: BDI, Beck Depression Inventory score; BMIc, body mass index categories (< 25, 25-30, ≥30); DHT, dihydrotestosterone; FSH, follicle stimulating hormone; LH, luteinising hormone; N, sample size; SHBG, sex hormone binding globulin  a Change (% change/ 4 years) in frailty index per standard deviation increase in hormone level. Negative % change means that the baseline hormone level is associated with improvement of frailty status and positive % change means that the hormone is associated with worsening frailty status | | | | | | | | | | | | | | | | | |

| **Supplemental Table 6. Multivariable-adjusted odds ratio (95% CI) for worsening frailty phenotype associated with baseline endocrine predictors** | | | | | | | | | | | | | | | | |
| --- | --- | --- | --- | --- | --- | --- | --- | --- | --- | --- | --- | --- | --- | --- | --- | --- |
| Models and adjustments | | | | | | | | | | | | | | | | |
| Baseline Parameter | N | Model 1  Baseline frailty | | | Model 2  Baseline frailty and age | | | Model 3  Baseline frailty, age, centre, smoking | | | Model 4  Baseline frailty, age, centre, smoking, BMIc | | | Model 5  Model 4+ CVD, DM | | |
|  |  | **OR** | **95% CI** | **P-value** | **OR** | **95% CI** | **P-value** | **OR** | **95% CI** | **P-value** | **OR** | **95% CI** | **P-value** | **OR** | **95% CI** | **P-value** |
| Total Testosterone | 1766 | 1.04 | 0.93, 1.16 | 0.446 | 1.04 | 0.93, 1.16 | 0.520 | 1.08 | 0.96, 1.22 | 0.187 | 1.05 | 0.92, 1.18 | 0.474 | 1.06 | 0.94, 1.20 | 0.343 |
| Free Testosterone | 1760 | 0.86 | 0.77, 0.96 | 0.008 | 0.98 | 0.87, 1.1 | 0.702 | 1.03 | 0.91, 1.16 | 0.681 | 0.99 | 0.88, 0.04 | 0.979 | 1.01 | 0.89, 1.15 | 0.872 |
| DHT | 1759 | 1.07 | 0.96, 1.19 | 0.213 | 1.03 | 0.92, 1.15 | 0.601 | 1.01 | 0.90, 1.14 | 0.822 | 0.96 | 0.85, 1.09 | 0.574 | 0.98 | 0.86, 1.11 | 0.770 |
| Estradiol* | 1759 | 1.04 | 0.94, 1.16 | 0.435 | 0.99 | 0.87, 1.13 | 0.894 | 1.07 | 0.94, 1.23 | 0.300 | 1.11 | 0.97, 1.28 | 0.136 | 1.11 | 0.96, 1.27 | 0.158 |
| SHBG | 1769 | 1.25 | 1.12, 1.39 | <.001 | 1.08 | 0.96, 1.22 | 0.181 | 1.10 | 0.97, 1.24 | 0.144 | 1.06 | 0.94, 1.21 | 0.342 | 1.07 | 0.94, 1.22 | 0.283 |
| FSH | 1768 | 1.21 | 1.09, 1.34 | <.001 | 1.09 | 0.98, 1.22 | 0.123 | 1.11 | 0.99, 1.24 | 0.085 | 1.10 | 0.98, 1.24 | 0.094 | 1.09 | 0.97, 1.23 | 0.137 |
| LH | 1767 | 1.20 | 1.08, 1.33 | 0.001 | 1.09 | 0.97, 1.22 | 0.132 | 1.08 | 0.96, 0.21 | 0.183 | 1.07 | 0.95, 1.20 | 0.257 | 1.06 | 0.95, 1.19 | 0.285 |
| * Models 2 - 5 additionally adjusted for baseline total testosterone level  Abbreviations: BMIc, body mass index categories (< 25, 25-30, ≥30); CVD, cardiovascular disease; DHT, dihydrotestosterone; DM, diabetes mellitus; FSH, follicle stimulating hormone; LH, luteinising hormone; N, sample size; SHBG, sex hormone binding globulin | | | | | | | | | | | | | | | | |

| **Supplemental Table 7. Multivariable-adjusted odds ratio (95% CI) for improving frailty phenotype associated with baseline endocrine predictors** | | | | | | | | | | | | | | | | |
| --- | --- | --- | --- | --- | --- | --- | --- | --- | --- | --- | --- | --- | --- | --- | --- | --- |
| Models and adjustments | | | | | | | | | | | | | | | | |
| Baseline Parameter | N | Model 1  Baseline frailty | | | Model 2  Baseline frailty and age | | | Model 3  Baseline frailty, age, centre, smoking | | | Model 4  Baseline frailty, age, centre, smoking, BMIc | | | Model 5  Model 4+ CVD, DM | | |
|  |  | **OR** | **95% CI** | **P-value** | **OR** | **95% CI** | **P-value** | **OR** | **95% CI** | **P-value** | **OR** | **95% CI** | **P-value** | **OR** | **95% CI** | **P-value** |
| Total Testosterone | 427 | 0.88 | 0.73, 1.06 | 0.181 | 0.85 | 0.69, 1.03 | 0.107 | 0.87 | 0.70, 1.07 | 0.183 | 0.87 | 0.69, 1.09 | 0.215 | 0.86 | 0.68, 1.08 | 0.194 |
| Free Testosterone | 427 | 1.09 | 0.90, 1.32 | 0.385 | 0.89 | 0.72, 1.10 | 0.290 | 0.89 | 0.71, 1.11 | 0.319 | 0.90 | 0.71, 1.13 | 0.349 | 0.90 | 0.71, 1.13 | 0.363 |
| DHT | 428 | 0.91 | 0.75, 1.10 | 0.322 | 0.92 | 0.75, 1.12 | 0.413 | 0.94 | 0.76, 1.16 | 0.589 | 0.96 | 0.77, 1.21 | 0.764 | 0.98 | 0.78, 1.24 | 0.896 |
| **Estradiol*** | 428 | 0.74 | 0.61, 0.89 | 0.002 | 0.81 | 0.64, 1.01 | 0.061 | 0.71 | 0.56, 0.91 | 0.008 | 0.68 | 0.52, 0.88 | 0.004 | 0.67 | 0.52, 0.88 | 0.004 |
| SHBG | 429 | 0.76 | 0.63, 0.92 | 0.006 | 0.88 | 0.72, 1.08 | 0.223 | 0.90 | 0.72, 1.12 | 0.343 | 0.91 | 0.72, 1.15 | 0.446 | 0.90 | 0.71, 1.15 | 0.401 |
| FSH | 429 | 0.88 | 0.76, 1.02 | 0.095 | 0.99 | 0.85, 1.15 | 0.906 | 1.02 | 0.88, 1.18 | 0.804 | 1.02 | 0.88, 1.19 | 0.791 | 1.04 | 0.90, 1.21 | 0.588 |
| LH | 428 | 0.80 | 0.67, 0.96 | 0.018 | 0.91 | 0.76, 1.09 | 0.302 | 0.94 | 0.79, 1.12 | 0.484 | 0.94 | 0.79, 1.12 | 0.492 | 0.96 | 0.80, 1.14 | 0.639 |
| * Models 2 - 5 additionally adjusted for baseline total testosterone level  Abbreviations: BMIc, body mass index categories (< 25, 25-30, ≥30); CVD, cardiovascular disease; DHT, dihydrotestosterone; DM, diabetes mellitus; FSH, follicle stimulating hormone; LH, luteinising hormone; N, sample size; SHBG, sex hormone binding globulin | | | | | | | | | | | | | | | | |


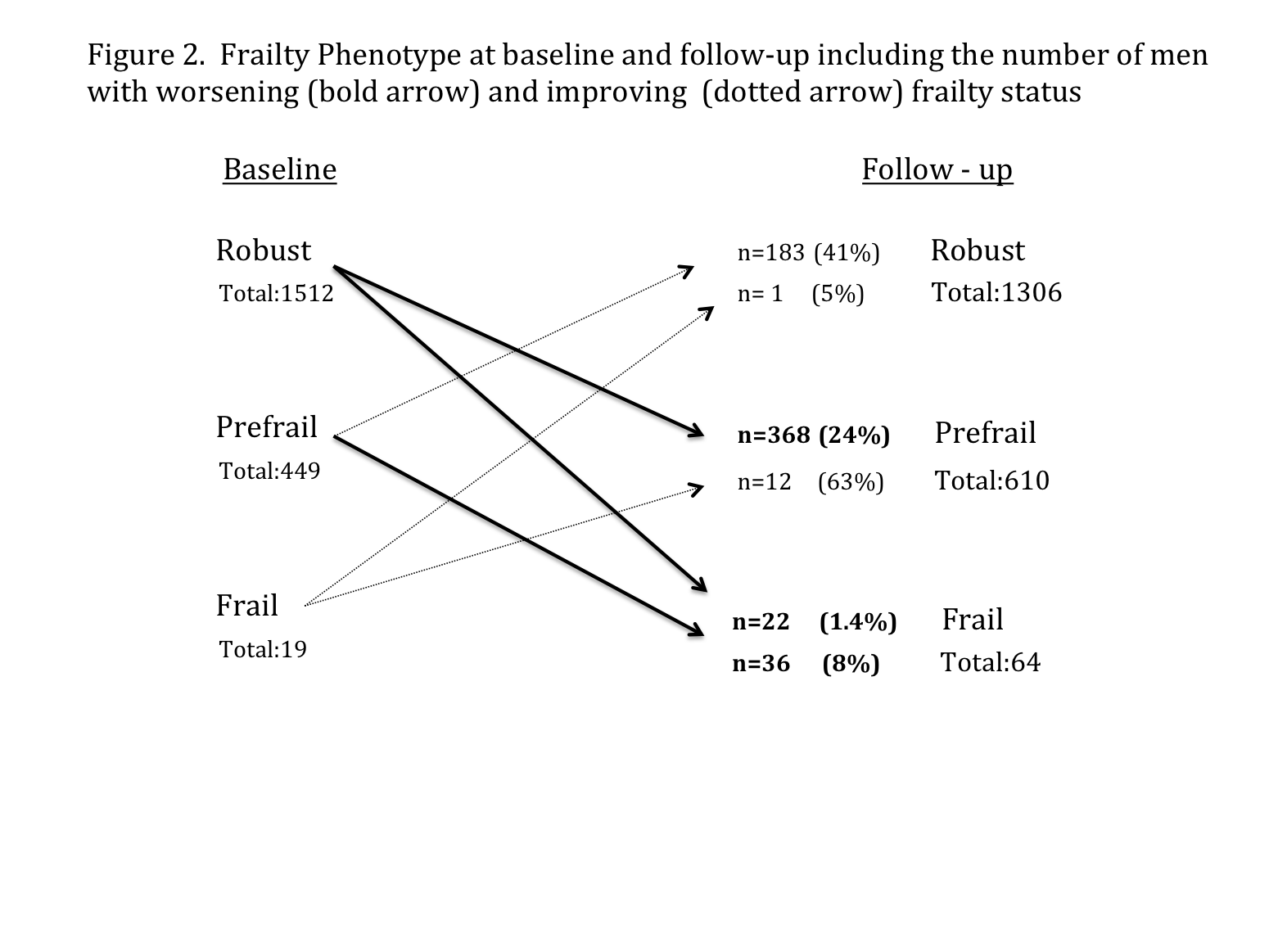


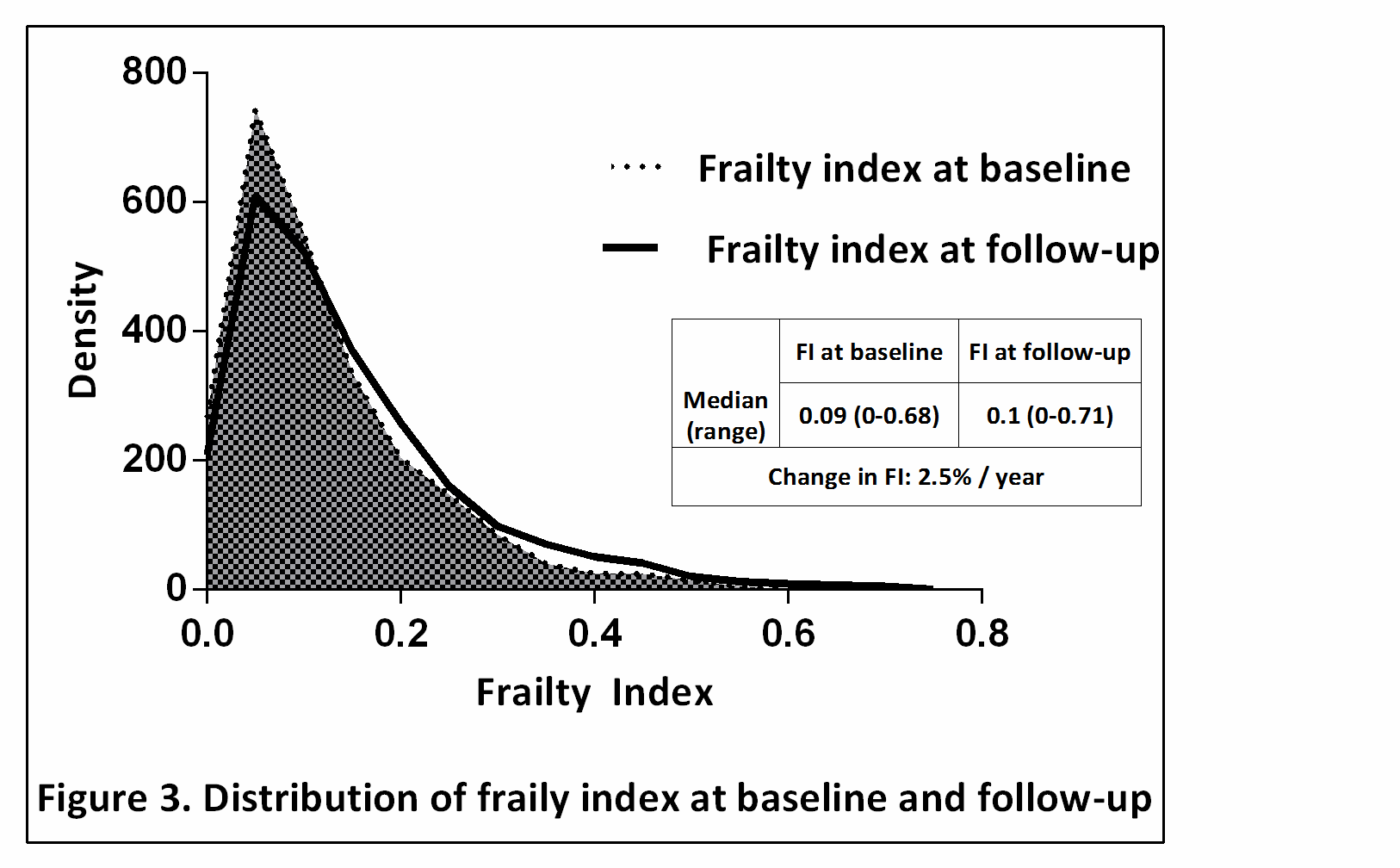


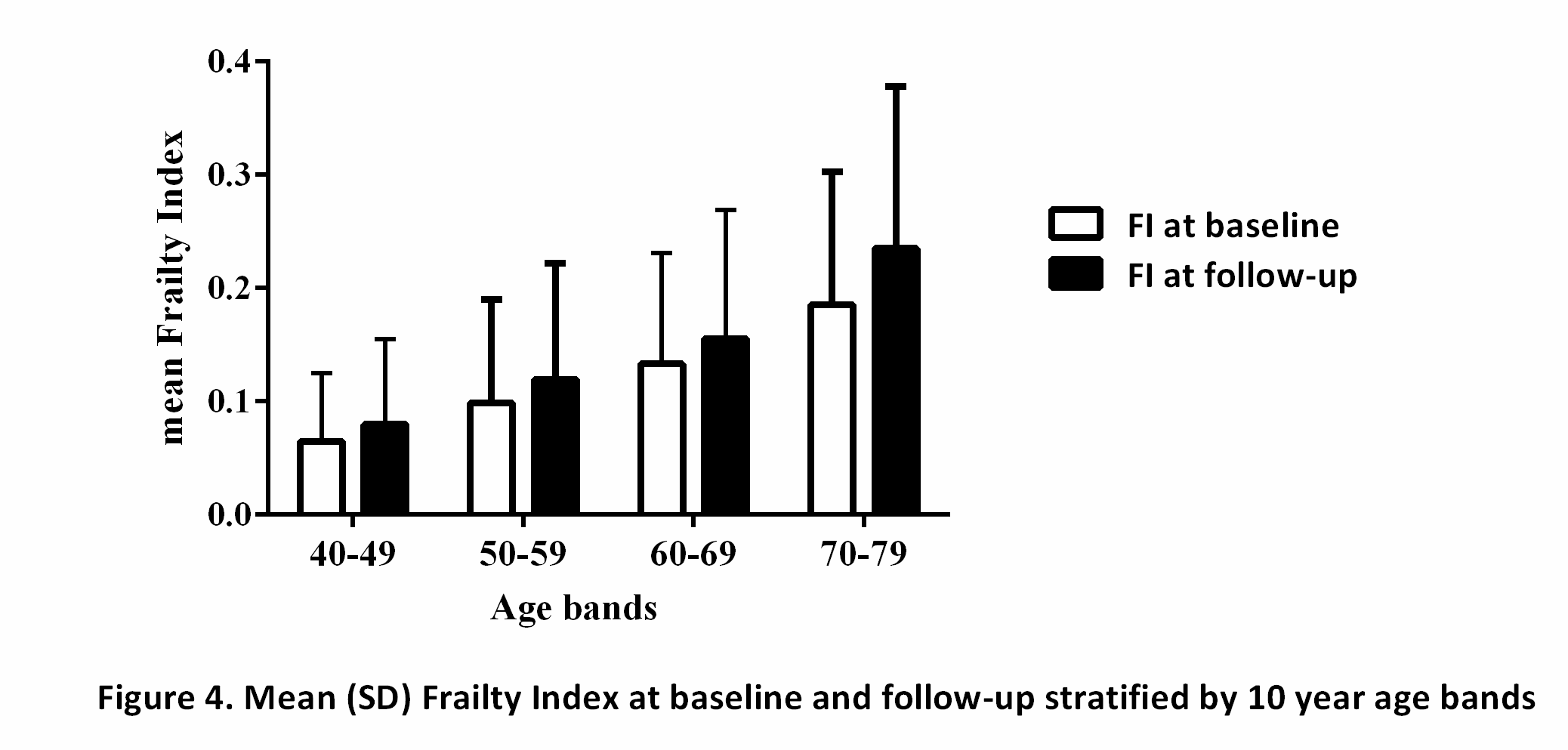


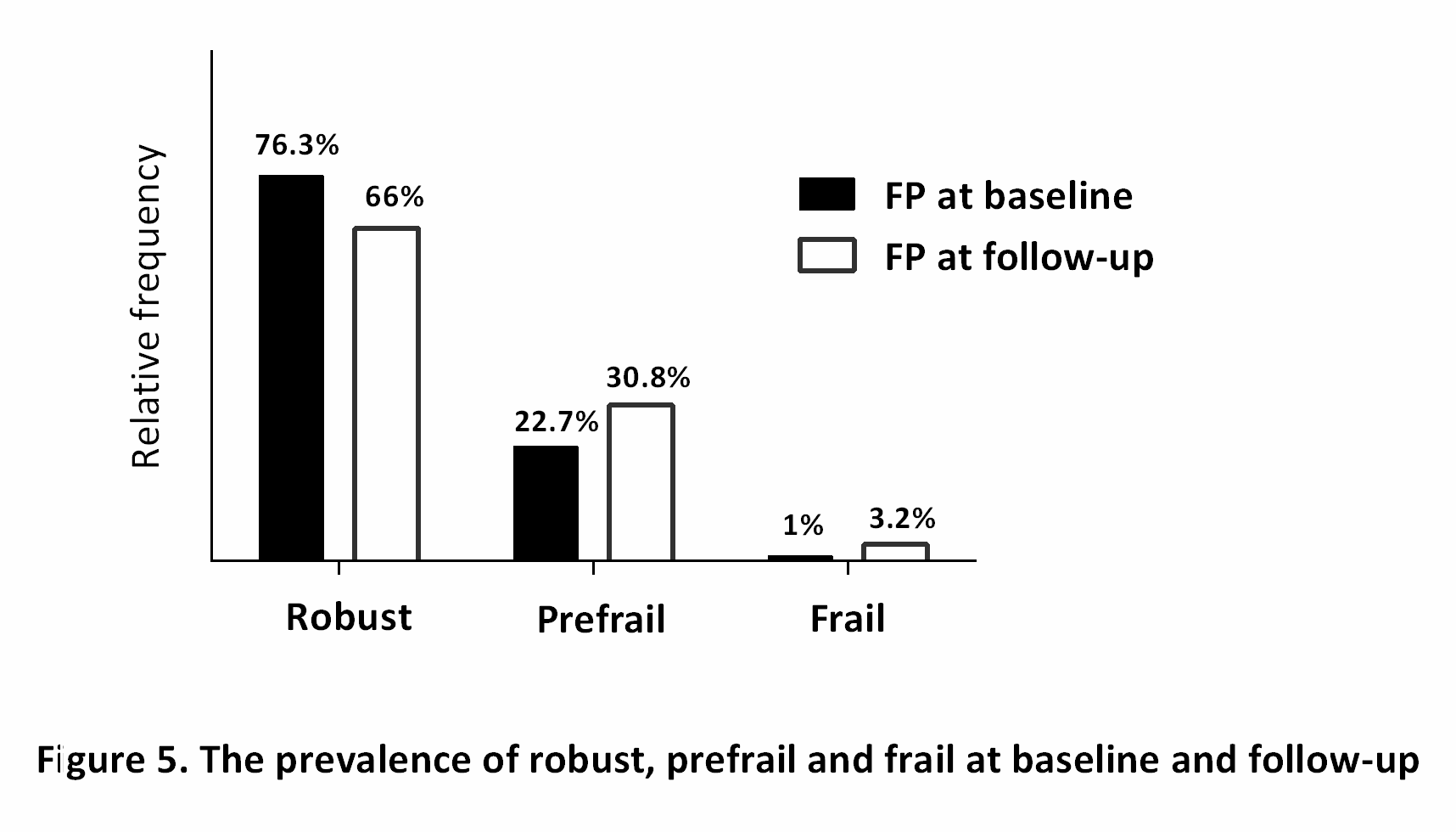

Supplement: Supplementary file 1 [file jc.2017-01172.st1.doc]
